# Spatial Learning Depends on Both the Addition and Removal of New Hippocampal Neurons 

**DOI:** 10.1371/journal.pbio.0050214

**Published:** 2007-08-07

**Authors:** David Dupret, Annabelle Fabre, Màtè Dàniel Döbrössy, Aude Panatier, José Julio Rodríguez, Stéphanie Lamarque, Valerie Lemaire, Stephane H. R Oliet, Pier-Vincenzo Piazza, Djoher Nora Abrous

**Affiliations:** 1 INSERM U862, Bordeaux Neuroscience Research Center, Bordeaux, France; 2 University of Bordeaux 2, Bordeaux, France; 3 The University of Manchester, Manchester, United Kingdom; Norwegian University of Science and Technology, Norway

## Abstract

The role of adult hippocampal neurogenesis in spatial learning remains a matter of debate. Here, we show that spatial learning modifies neurogenesis by inducing a cascade of events that resembles the selective stabilization process characterizing development. Learning promotes survival of relatively mature neurons, apoptosis of more immature cells, and finally, proliferation of neural precursors. These are three interrelated events mediating learning. Thus, blocking apoptosis impairs memory and inhibits learning-induced cell survival and cell proliferation. In conclusion, during learning, similar to the selective stabilization process, neuronal networks are sculpted by a tightly regulated selection and suppression of different populations of newly born neurons.

## Author Summary

The birth of adult hippocampal neurons is associated with enhanced learning and memory performance. In particular, spatial learning increases the survival and the proliferation of newborn cells, but surprisingly, it also decreases their number. Here, we hypothesized that spatial learning also depends upon the death of newborn hippocampal neurons. We examined the effect of spatial learning in the water maze on cell birth and death in the rodent hippocampus. We then determined the influence of an inhibitor of cell death on memory abilities and learning-induced changes in cell death, cell proliferation, and cell survival. We show that learning increases the elimination of the youngest newborn cells during a specific developmental period. The cell-death inhibitor impairs memory abilities and blocks the learning-induced cell death, the survival-promoting effect of learning on older newly born neurons, and the subsequent learning-induced proliferation of neural precursors. These results show that spatial learning induces cell death in the hippocampus, a phenomenon that subserves learning and is necessary for both the survival of older newly born neurons and the proliferation of neural precursors. These findings suggest that during learning, neuronal networks are sculpted by a tightly regulated selection of newly born neurons and reveal a novel mechanism mediating learning and memory in the adult brain.

## Abstract 3

Spatial learning-induced cell death in the hippocampus is necessary for the survival of newly born cells, the proliferation of neural precursors, and the retention of spatial memories.

## Introduction

It was classically assumed that once the development of the central nervous system ended, “everything can die, nothing can regenerate and be renewed” [1]. This dogma, restricting neurogenesis to a developmental phenomenon has, however, been challenged by the discovery that new neurons are created in specific regions of the adult mammalian brain [2,3]. The dentate gyrus (DG) of the hippocampal formation is one of the few structures where adult neurogenesis occurs in mammals [4], and it has been estimated that several thousand new cells are generated daily [5,6]. Neurogenesis in the DG is a complex, multistep process that starts with the proliferation of neural precursors residing in the dentate subgranular layer. Within a few days following their birth, at least 50% of the daughter cells die [7]. The adult-born cells that survive this initial period of cell death differentiate, for the most part into granule neurons, and survive for several months within the DG [8]. These new mature neurons receive synaptic inputs, extend axons along the mossy fiber tract, and exhibit electrophysiological properties very similar to those of mature dentate granule neurons [9–12].

The involvement of the hippocampal formation in memory has long been recognized [13], and increasing evidence suggests that the production of adult-born neurons may contribute to memory processes. First, the rate of neurogenesis is positively correlated to hippocampal-mediated learning abilities [14]. Second, conditions that increase memory performance enhance neurogenesis, whereas conditions that decrease neurogenesis impair learning [15–18]. Third, spatial learning has been shown to increase both the survival of newborn neurons [19] and cell proliferation [16,20]. Remarkably, spatial learning in a water maze was also linked to a decrease in the number of newborn neurons in the DG [20,21]. Even more surprising, the decline in neurogenesis is correlated with spatial abilities, i.e., rats with the lowest number of newly born cells have the best memory performances, indicating that learning, and not training, decreased the number of adult-born cells [20].

These complex results provide a puzzling picture in which increases and decreases in the number of newborn neurons are both correlated with learning. In order to solve this discrepancy, we hypothesized that spatial learning is accompanied by events that are similar to the selective stabilization process observed during development. Indeed, during brain development, many more neurons are produced than are actually needed, and the active and selective removal of the cells that have not yet established appropriate synaptic connections allows for the sculpting of the relevant and functional neural networks. In this study, we found that learning has three effects on neurogenesis. Learning promotes the survival of relatively mature neurons, induces the death of more immature neurons, and finally, stimulates cell proliferation. Cell death seems to be a pivotal event in this cascade because blocking learning-induced apoptosis inhibits the other two cellular events and impairs memory abilities. These results indicate that spatial learning involves a cascade of events similar to the selective stabilization process by which neuronal networks are sculpted by adding and removing specific population of cells as a function of their maturity and functional relevance.

## Results

### Specific Phase of Spatial Learning Induces Apoptotic Cell Death in the Dentate Gyrus

In these experiments, rats were trained in a water maze, one of the most commonly used tests for spatial learning in rodents. In this task, the animals learn across daily sessions to find a hidden escape platform using the distal cues present in the surrounding environment.

**Figure 1:**
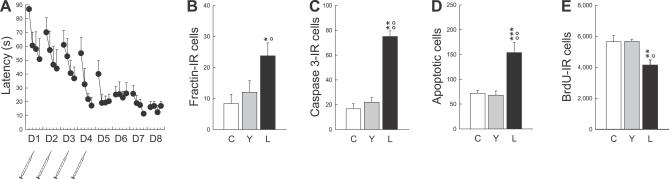
Spatial Learning in a Water Maze Increases Cell Death and Decreases Cell Genesis in the Dentate Gyrus (A) Latency to find the escape platform. The syringes represent BrdU injections. (B–D) Apoptotic cell death measured by the number of fractin-IR cells (B), active-caspase-3-IR cells (C), and both pyknotic and karyorrhexic dying cells (Apoptotic cells) (D). (E) Cell genesis measured by the number of BrdU-IR cells. C, Control group; D, day; L, Learning group; Y, Yoked group. A single asterisk (*) indicates *p ≤* 0.05, double asterisks (**) indicate *p* ≤ 0.01, and triple asterisks (***) indicate *p* ≤ 0.001 compared to Control. A single circle (°) indicates *p* ≤ 0.05, double circles (°°) indicate *p* ≤ 0.01 compared to Yoked. Error bars represent the standard error of the mean (s.e.m.).

**Figure 2:**
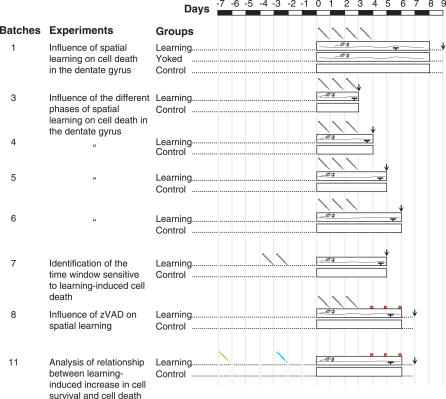
Schematic Representation of the Main Experiment Syringes represent XdU BrdU (gray), IdU (green), and CldU (blue) injections. Black arrows represent the time of sacrifice. Red squares represent intracerebroventricular infusions (zVAD or Vehicle).

**Figure 3:**
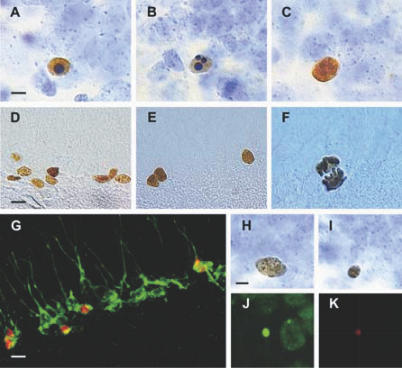
Representative Examples of Apoptotic, Newborn, and Proliferative Cells (A) Fractin-IR pyknotic cell. (B) Fractin-IR karyorrhexic cell. (C) Active-caspase-3-IR cell. (D) BrdU-IR cells. (E) Ki67-IR cells. (F) HH3-IR cell in anaphase. (G) BrdU-labeled cells (red) are stained with Dcx (green), a typical marker for immature newborn neurons. (H) Healthy BrdU-DAB-IR cell. (I) Pyknotic BrdU-DAB-IR cell. (J and K) Small BrdU-IR cell (red) exhibiting nuclear shrinkage using Fluoro Nissl Green counterstaining. Bar scales: (A–C), (F), and (H–K) indicate 5 μm; (D), (E), and (G) indicate 10 μm.

In a first experiment (Figure 1A), animals trained in the water maze (Learning group [L]) were compared to two other groups. The first group was composed of animals that were transferred to the testing room at the same time and with the same procedures as the learning group except that they were not exposed to the water maze (Control group [C]). The second group (Yoked group [Y]) contained animals that were submitted to the same procedures as the control group except that they were also exposed to the water maze for a time period equivalent to that of the Learning group but in the absence of the escape platform. Control and Yoked animals allowed us to control for the putative influence of stress and physical exercise (batch 1, see Figure 2 and Table S1). At the end of the training, cell death was assessed in these three experimental groups by using two specific apoptotic markers, the active form of caspase 3 and the caspase 3–cleaved fragment of actin called fractin (Figure 3A–3C) [22]. Pyknosis and karyorrhexis were also used as morphological criteria to evaluate apoptotic cell death (Figure 3A and 3B).

It was found that spatial learning induced apoptotic cell death in the DG. Thus, although Control and Yoked animals did not differ in the number of apoptotic cells, a higher number was found in the Learning group (Figure 1B, *F*_2,16_ = 4.70, *p* < 0.05; Figure 1C, *F*_2,16_ = 11.01, *p* < 0.001; and Figure 1D, *F*_2,16_ = 12.90, *p* < 0.001). Learning-induced apoptosis was associated with a decrease in the number of newly born cells that were produced during the early phase of training (Figures 1E and 3D, *F*_2,16_ =7.38, *p* ≤ 0.01). Newly born cells were identified by injecting animals during the first 4 d of training with 5-bromo-2′-deoxyuridine (BrdU). Consistent with these findings, learning-induced cell death occurred prominently in the subgranular layer, which is the part of the DG where neuronal precursors and immature neurons reside (Table S2). Furthermore, learning-induced cell death was specific to the DG; no changes were observed in the CA3 and CA1 regions of the Ammon's Horn of the hippocampus (Table S3).

In a second experiment, we determined whether learning a task that does not require the hippocampus also increases cell death [23]. To this end, a fourth group of animals trained to find a visible platform (Visible platform group [VP]) were compared to the Control, Yoked, and Learning groups (batch 2). We found that cell death was specific to spatial learning. Thus, we found an increase in apoptotic cell death only in the Learning group, whereas the Visible platform, Control, and Yoked groups did not differ (Control group = 65.00 ± 11.73 cells, Yoked group = 54.00 ± 8.12 cells, Visible platform group = 50.00 ± 5.92 cells, Learning group = 97.00 ± 9.03 cells, *F*_3,16_=5.67, *p* < 0.001; Control group = Yoked group = Visible platform group < Learning group, all comparisons at least *p* ≤ 0.01).

**Figure 4:**
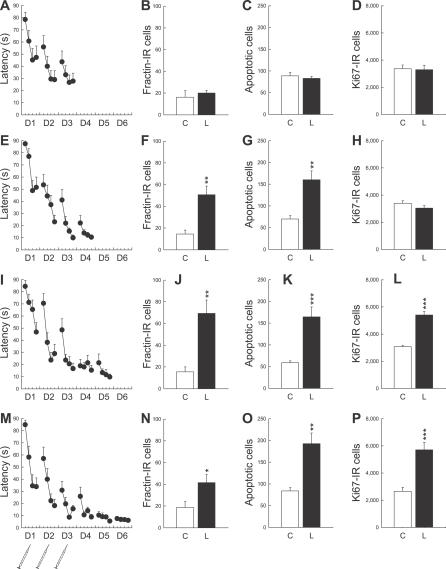
The Stabilization of Spatial Performances Increases Cell Death and Cell Proliferation in the Dentate Gyrus (A, E, I, and M) Latency to find the escape platform. The syringes represent BrdU injections. D, day. (B, F, J, and N) Apoptosis measured by the number of fractin-IR cells. (C, G, K, and O) Apoptosis measured by the number of both pyknotic and karyorrhexic dying cells. (D, H, L, and P) Cell proliferation measured by the number of Ki67-IR cells. A single asterisk (*) indicates *p* ≤0.05, double asterisks (**) indicate *p* ≤ 0.01, and triple asterisks (***) indicate *p* ≤ 0.001 compared to Control groups. Error bars represent the s.e.m.

In a third experiment, we then examined whether a particular phase of the learning process was responsible for the induction of apoptosis (batches 3–6). Indeed, during training in the water maze, two phases can be distinguished: an early phase during which performance improves rapidly, and a late phase during which performance stabilizes, reaching an asymptotic level [20]. In order to distinguish the effects of these two phases of learning on apoptosis, independent Control and Learning groups were sacrificed at different days of training (Figure 4A, 4E, 4I, and 4M) starting at day 3. Since in the two previous experiments the Control and Yoked animals did not differ, only the Control group was used for this and subsequent experiments.

It was found that only the asymptotic phase of learning induced cell death. Thus, an increase in cell death was seen starting from the fourth day of training (Figure 4F, 4G, 4J, 4K, 4N, and 4O), which corresponds to the beginning of the asymptotic phase, but not at day 3, which corresponds to the early phase (Figure 4B and 4C). In order to further characterize the relationship between learning and changes in cell death, we correlated performance in the water maze with the number of apoptotic cells (Figure S1). A positive relationship was found; the animals that had the best learning of the task (lower latency to reach the platform) also had the highest number of dying cells.

Finally in this experiment, we studied the effects of the different phases of learning on cell proliferation and on the number of newborn cells produced during the early phase of learning. Cell proliferation was analyzed by studying the expression of Ki67 [4], a nuclear protein expressed for the entire duration of the cell cycle (Figures 3E, 4D, 4H, 4L, and 4P). We found that the asymptotic phase of learning stimulates the production of new cells. However, this phenomenon appears 1 d after the start of cell death, i.e., at the fifth day of training instead of the fourth (Figure 4L, *t*_21_ = 7.02, *p* < 0.001; and Figure 4P, *t*_19_ = 4.06, *p* < 0.001). In contrast**,** in this experiment learning had no effect on the number of newborn cells labeled with BrdU during the first 3 d of training (Table S4).

### Spatial Learning Promotes the Death of Newborn Neurons within a Certain Time Window

The results of the previous experiments seem contradictory concerning the decrease in the number of newborn neurons. Thus, this phenomenon was observed during the first experiment but not the third. One possible explanation for this discrepancy is the difference in the age of the newly born neurons that were studied in the two experiments. In the first experiment (batch 1), in which a decrease in newborn neurons was found, BrdU-labeled cells were between 5 and 8 d old at the time of the sacrifice (Figure 2). In contrast, in the third experiment (batches 3–6), in which learning did not decrease the numbers of newly born neurons, BrdU-immunoreactive (IR) cells at the time of the sacrifice were less than 5 d old (Figure 2).

**Figure 5:**
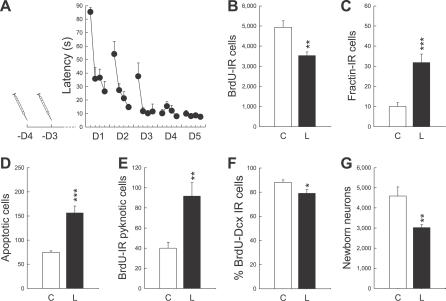
Spatial Learning Promotes the Death of Newborn Neurons Generated a Few Days before Training (A) Latency to find the escape platform. Learning groups were injected with BrdU either 4 or 3 d before initiation of training as indicated by the syringes. D, day. (B) Newly born cells measured by the number of BrdU-IR cells. (C and D) Apoptotic cells measured by the number of fractin-IR cells (C) and both pyknotic and karyorrhexic dying cells (D). (E) Number of BrdU-IR cells exhibiting characteristics of dying cells. (F) Percentage of BrdU-IR cells expressing the immature neuronal marker Dcx. (G) Extrapolated number of newborn neurons. A single asterisk (*) indicates *p* ≤ 0.05, double asterisks (**) indicate *p* ≤ 0.01, and triple asterisks (***) indicate *p* ≤ 0.001 compared to Control groups. Error bars represent the s.e.m.

These data suggest that learning promotes the death of cells that have reached a certain level of maturation and are older than 5 d. To test this hypothesis, additional groups of animals were trained in the water maze under conditions similar to those of experiment 3, but were injected with BrdU either 3 or 4 d before the start of the behavioral training (batches 7a and 7b, Figure 5A). In this way, BrdU-labeled cells would be either 7 or 8 d old at the end of the 5 d of training (Figure 2). We found that for both ages of BrdU-labeled cells, learning induced a decrease in the number of newly born cells (Figure 5B, group × age interaction *F*_1,20_ = 0.01, *p* = 0.90; group effect: *F*_1,20_ = 12.57, *p* < 0.01) and, as expected, an increase in cell death (Figure 5C, group × age interaction *F*_1,20_ = 2.82, *p* = 0.11; group effect: *F*_1,20_ = 27.34, *p* < 0.001; and Figure 5D, group × age interaction *F*_1,20_ = 1.83, *p* = 0.19; group effect: *F*_1,20_ = 35.78, *p* < 0.001).

To confirm that this decrease in the number of BrdU-labeled cells was due to learning-induced death of newly born neurons, we first evaluated the number of BrdU-labeled cells that expressed morphological manifestations of apoptosis. In comparison to healthy BrdU-IR cells with large nuclei (≈10 μm, Figure 3H), apoptotic newly born BrdU-labeled cells exhibited a small round shape and condensed, shrunken nuclei (≈3–4 μm, Figure 3I–3K). It was found that learning increased the number of BrdU-IR pyknotic cells (Figure 5E, *t*_12_ = −3.83, *p* ≤ 0.01). We then studied the percentage of BrdU-labeled cells expressing the immature neuronal marker doublecortin (Dcx). It was found that this population of cells was also decreased, most probably because the dying BrdU-labeled cells lost the Dcx labeling (Figures 3G and 5F, *t*_8_ = −2.30, *p* ≤ 0.05). In addition, a quantitative evaluation based on the extrapolated total number of BrdU-labeled neurons showed that learning induces a decrease of 34% in this population of cells, which corresponds to a loss of approximately 1,600 newborn neurons (Figure 5G, *t*_8_ = −3.31, *p* ≤ 0.01).

### Learning-Induced Apoptosis Is Critical for Spatial Memory

**Figure 6:**
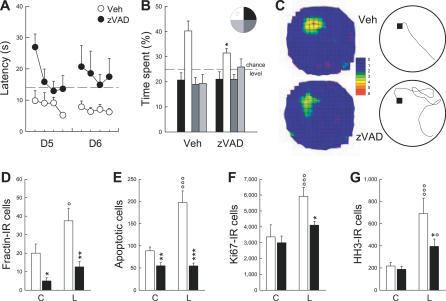
zVAD Infusion Blocks Learning-Induced Cell Death and Impedes Spatial Memory (A) Latency to reach the hidden platform in animals infused with zVAD (•) or vehicle (○). The dashed line represents the mean escape latency across the first four training days. (B) Memory of platform location during a probe test (seventh day of testing) measured by the time spent in the target quadrant (open bar). A single asterisk (*) indicates *p* ≤ 0.05. (C) Spatial histograms of the animals' locations and examples of swim paths to reach the platform location during the probe test (see Protocol S1 for more information). (D and E) Effects of zVAD (filled bars) and vehicle (open bars) treatments on cell death measured by the number of fractin-IR cells (D) and both pyknotic and karyorrhexic dying cells (E). (F and G) Effects of zVAD (filled bars) and vehicle (open bars) treatments on cell proliferation measured by the number of Ki67-IR cells (F) and HH3-IR cells (G). A single asterisk (*) indicates *p* ≤ 0.05, double asterisks (**) indicate *p* ≤ 0.01, and triple asterisks (***) indicate *p* ≤ 0.001, zVAD group compared to Veh group; a single circle (°) indicates *p* ≤ 0.05, and triple circles (°°°) indicate *p* ≤ 0.001, Learning group (L) compared to Control group (C). Error bars represent the s.e.m

We then examined whether learning-induced cell death plays a role in the stabilization of performances in the water maze. To address this issue, at the end of the fourth training session, i.e., when learning-induced cell death becomes apparent, animals were infused in the lateral ventricles with either the pan-caspase inhibitor z-Val-Ala-Asp-fluoromethylketone (zVAD) or with vehicle [24,25] (batch 8). These treatments were repeated for two subsequent training days. Before the beginning of the treatment, the vehicle- and zVAD-infused animals did not differ in their latency to find the hidden platform (unpublished data; *F*_9,198_ = 1.16, *p* = 0.32). However, after the infusions, zVAD-infused animals were significantly impaired, whereas vehicle-infused rats continued to stabilize their performances (Figure 6A, *F*_1,22_ = 15.64, *p* < 0.001). This was particularly obvious in the first trial on day 5, during which zVAD-infused animals exhibited the largest impairment, suggesting a deficit in retrieving what was learned on the preceding day. In contrast, zVAD-treated animals were able to learn, within the session, the position of the hidden platform. These data strongly suggest that inhibition of apoptosis disrupts the memory trace and not learning per se.

To further evaluate the strength of the memory trace after zVAD treatment, a probe test was performed on the seventh day of training. The probe test consisted of exposing animals to the water maze in the absence of the escape platform and recording the time spent in the quadrant of the water maze that contained the platform during training (target quadrant). zVAD-infused animals spent less time than vehicle rats in the target quadrant (Figure 6B and 6C, *t*_22_ = 2.01, *p* ≤ 0.05). In addition, several indices used to measure the efficiency of the swim paths to reach the goal location were also impaired in zVAD-infused animals (Table S5). These results confirm that the inhibition of apoptosis when animals begin to master the task leads to an impairment of the memory for the platform location.

We found that zVAD treatment efficiently prevented apoptosis (Figure 6D, *F*_3,39_ = 9.61, *p* < 0.001; and Figure 6E, *F*_3,39_ = 21.23, *p* < 0.001). However, we also found that zVAD treatment greatly reduced the increase in cell proliferation induced by the late phase of learning. In this experiment, cell proliferation was measured by Ki67 staining (Figure 6F, *F*_3,39_ = 6.72, *p* < 0.001), and also by a second marker of cell genesis, the phosphorylated histone H3 [4] (HH3, Figures 3F and 6G, *F*_3,39_ = 6.89, *p* < 0.001). These results indicate that the increase in the production of new cells observed during the asymptotic phase of learning probably constitutes a compensatory phenomenon triggered by learning-induced apoptosis. Thus, as shown in experiment 3 (Figure 4), learning-induced cell proliferation follows learning-induced cell death and, as shown in the present experiment, this phenomenon is decreased by blocking cell death.

In order to test for the specificity of the effects of zVAD, we performed several complementary measures and experiments. Thus, after the probe test, animals were tested for their ability to find a visible platform (cued test), which allowed for a measure of visuomotor processes (Table S5). In this case, zVAD treatment had no measurable effects on behavioral performance. We also evaluated whether zVAD would modify the survival of newly born cells younger than 5 d, because these neurons are normally untouched by learning. This population of cells was labeled by injecting BrdU on day 1 to day 3 of training (Figure 2). Again, zVAD treatment had no measurable effects (*t*_22_ = 0.87, *p* = 0.39). Then, in a subsequent experiment (batch 9), we infused zVAD during the first 3 d of training, a period during which learning has no influence on cell death (Figure 4B and 4C). Consequently, if the effects of zVAD are mediated by a blockade of learning-induced cell death, this schedule of treatment should have no behavioral effects. Indeed, we found that under these conditions, zVAD infusions did not impair spatial memory (Figure S2). Finally, we performed physiological recordings in the Ammon's horn, a part of the hippocampus in which learning does not induce cell death (Table S3) and which consequently should not respond to zVAD treatment (batch 10). Field recordings confirmed that zVAD did not alter excitatory synaptic transmission within this area (Figure S3).

In conclusion, taken together, all these control experiments confirm that the disruption of spatial learning was due to a specific inhibition of apoptosis by zVAD.

### Learning-Induced Increases in Apoptosis, Cell Proliferation, and Survival of Newborn Neurons Are Interrelated Processes

**Figure 7:**
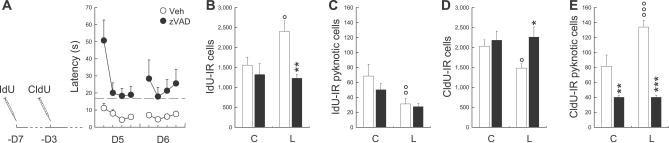
Learning Increases the Number of New Neurons, and This Effect Is Blocked by zVAD Infusion (A) Latency to reach the hidden platform in animals infused with zVAD (•) or vehicle (○). The dashed line represents the mean escape latency across the first four training days. The syringes represent IdU and CldU injections. D, day. (B) Effects of zVAD (filled bars) and vehicle (open bars) treatments on the survival of IdU-IR cells generated 7 d before exposure to the task. (C) Effects of zVAD (filled bars) and vehicle (open bars) treatments on the number of IdU-IR cells exhibiting characteristics of pyknotic cells. (D) Effects of zVAD (filled bars) and vehicle (open bars) treatments on the survival of CldU-IR cells generated 3 d before exposure to the task. (E) Effects of zVAD (filled bars) and vehicle (open bars) treatments on the number of CldU-IR cells exhibiting characteristics of pyknotic cells. A single asterisk (*) indicates *p* ≤ 0.05, double asterisks (**) indicate *p* ≤ 0.01, and triple asterisks (***) indicate *p* ≤ 0.001, zVAD group compared to Veh group; a single circle (°) indicates *p* ≤ 0.05, double circles (°°) indicate *p* ≤ 0.01, and triple circles (°°°) indicate *p* ≤ 0.001, Learning group (L) compared to Control group (C) .

It has previously been shown that training in a water maze increases the survival of newborn neurons that were produced 1 wk before the start of the training [19,26]. It seems then that learning can induce increases in both the survival and death of newborn neurons. For this reason, in a final experiment, we studied the relationships between these two phenomena (batch 11). This experiment was performed by injecting the same animals with two thymidine analogs, 5-iodo-2′-deoxyuridine (IdU) and 5-chloro-2′-deoxyuridine (CldU) [27] (Figure S4). IdU and CldU were injected at different times in order to analyze in the same subject the fate of newborn cells of different ages (Figure 2). IdU was injected 7 d before training in order to label the newborn cells for which survival should be increased by learning. CldU was injected 3 d before the start of the training in order to label newly born cells that should die as a consequence of learning. Animals in these experiments were also infused either with vehicle or zVAD at the end of the fourth through sixth days of training. As found in the previous experiment, vehicle- and zVAD-infused animals did not differ in their latency to find the hidden platform during the first 4 d of training (*F*_9_,_135_ = 0.52, *p* > 0.05). Similarly, an impairment was observed in zVAD-infused animals during the last two training days (Figure 7A, *F*_1_,_15_ = 20.43, *p* < 0.001).

In vehicle-treated rats, learning promoted the survival of IdU-labeled cells generated 1 wk before exposure to the task, and this effect was blocked by zVAD infusion (Figure 7B, *F*_3,24_ = 6.65, *p* < 0.01). This prosurvival effect of learning on IdU-labeled cells was associated with a decrease in the number of IdU-IR pyknotic cells (Figure 7C, *F*_3,24_ = 5.58, *p* < 0.01). Furthermore, as previously found here (Figure 5B), learning decreased the survival of CldU-labeled cells that were born 3 d before the start of the training, an effect suppressed by zVAD infusion (Figure 7D, *F*_3,24_ = 4.50, *p* ≤ 0.01). As expected, learning increased the number of CldU-IR pyknotic cells, and this effect was blocked by zVAD infusion (Figure 7E, *F*_3,24_ = 40.55, *p* ≤ 0.001). These data show that learning-induced increases in survival and apoptosis of newborn cells are interrelated processes. Thus, blocking learning-induced apoptosis also blocks the increased survival of older neurons.

## Discussion

The results of the experiments reported here show that spatial learning promotes the survival of adult-born neurons that are relatively more mature, induces the death of cells that are more immature, and finally, stimulates proliferation of precursors. Blocking learning-induced cell death has shown an interdependency of these events and their involvement in learning. Thus, blocking learning-induced apoptosis inhibits cell survival and cell proliferation, and impairs memory abilities. These results indicate that spatial learning could involve a cascade of events similar to the selective stabilization process by which neuronal networks are sculpted by adding and removing specific populations of cells as a function of their maturity and functional relevance.

Learning-induced apoptosis is a very specific phenomenon. It is selectively induced by a specific phase of spatial learning, the late phase, during which performances stabilize. In contrast, apoptosis of newborn neurons does not seem to be influenced by stress and/or physical activity: (1) animals were habituated to the pool before training in order to diminish its stressful component (but see also [28]); (2) learning did not induce cell death during the first 3 d of training, during which physical activity is at its highest; and (3) no modification in apoptosis was observed in Yoked animals exposed to the pool for 6 or 8 d. Furthermore, apoptosis was not influenced by hippocampus-independent learning in the water maze, such as cued learning of the platform position. Finally, the learning-induced increase in cell death is correlated with spatial abilities, i.e., rats with the highest number of dying cells have the best memory performances. This observation confirms that spatial learning, and not training, physical activity, or stress, increases apoptosis.

Spatial learning-induced apoptosis targets a population of young newborn neurons that are within a specific time window. Indeed, learning did not promote the death of newly born cells that were younger than 5 d or older than 13 d at the time of the sacrifice. In contrast, it promotes the death of cells that are 7 and 9 d old at the time of the sacrifice. These results are consistent with recent studies showing that the selective regulation of survival/death by input activity or the response to experience-specific modifications of adult-born neurons occur at a critical period during an immature stage [29,30].

We also showed that the administration of the antiapoptotic agent zVAD induces deficits in spatial memory. This is consistent with an earlier observation showing that administration of anti-caspases impaired spatial memory [31]. Here, we show that spatial memory impairment after caspase inhibition is due to the blockade of learning-induced neuronal apoptosis. The implication of apoptosis in learning seems quite specific. Thus, when the caspase inhibitor zVAD was infused during learning, but outside the window of learning-induced apoptosis, no effects on spatial learning were observed. In addition, zVAD injections per se did not alter the neurophysiological responsiveness of the hippocampus in a non-neurogenic area. Altogether, these data show that it is the learning-induced apoptosis in the DG that is involved in spatial memory.

**Figure 8:**
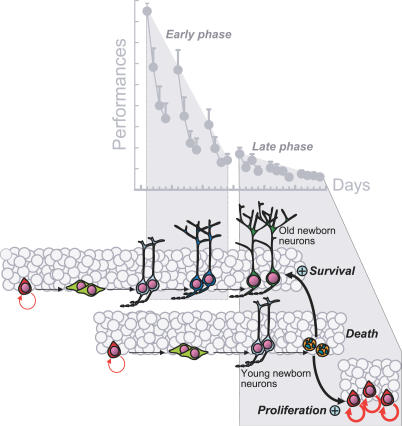
Spatial Learning Depends upon a Selective Stabilization Process Where the Production of New Neurons Is Followed by an Active and Selective Removal of Others The early phase of learning in the water maze, characterized by a fast improvement in performance, increases the survival of newborn neurons that were produced 1 wk before exposure to the task. Once the task has begun to be mastered, during the late phase of learning, learning induces apoptosis of newborn neurons that are a few days younger than the ones for which survival has been increased. This wave of cell death is followed by an increase in cell proliferation. Learning-induced apoptosis plays a pivotal role in this intermingled chain of events since it is necessary for the survival of the older, newly born neurons, but also for the increase in cell proliferation occurring during the late phase of learning.

The relationship described here between learning-induced increases in survival, apoptosis, and proliferation of newborn cells provides a three-step picture of the relationship between neurogenesis and spatial learning (Figure 8). First, acquisition of the task induces an increase in the survival of newborn neurons generated 1 wk before the task and that consequently have reached an intermediate level of maturity. Second, once the task starts to be mastered, learning induces apoptosis of newborn neurons that are a few days younger than those for which survival has been increased. Third, learning-induced apoptosis is followed by an increase in cell proliferation that provides the hippocampus with a new pool of young neurons [16,20].

This homeostatic regulation of neurogenesis by learning is consistent with the selective stabilization theory according to which regressive events will stabilize a particular set of contacts among many others, thereby sculpting the precise circuits that are crucial for a given function [32]. It has been estimated that during development, after an initial proliferating phase during which a large number of newborn neurons are produced, at least half of the initial neuronal population is eliminated by apoptosis [33]. This neuronal elimination serves several functions, among which is the regulation of target innervation. Indeed, neural function depends upon a precise quantitative relationship between neurons: each axon innervates an appropriate number of target cells and each target cell is innervated by an appropriate number of axons. The decision for survival or death during development is governed by afferences and/or efferences [34,35].

In the case of hippocampal adult-born neurons, it might be hypothesized that those cells that are successfully connected, both in terms of efferent output and afferent input, are the ones that can be rescued by the stimuli generated in the course of learning. In favor of this hypothesis, it has been shown that enhanced synaptic activity enhances cell survival [36]. In contrast, apoptosis could constitute a trimming mechanism that suppresses more-immature neurons that have not been selected by learning. Their suppression could favor the integration of older cells that have been stabilized by activity-dependent stimuli generated in the course of learning. These regressive events could also, by clearing the network of nonspecific noise due to superfluous new neurons, enhance the signal-to-noise ratio. Supporting this idea, an improvement in the signal-to-noise ratio of motor cortex cells during motor skill learning has been linked to a practice-related improvement in behavioral performance [37].

The precise mechanisms by which learning promotes the survival or apoptosis of immature newborn neurons are currently unknown. However, analysis of the developmental pattern of newborn neurons provides a certain number of putative explanations. Newborn neurons follow a precise maturation of neuronal connectivity and function that requires about 1 mo. They extend their dendritic tree at variable times after mitosis, and by 3 wk, their dendritic arborization resembles that of mature neurons [12,38]. In addition, as soon as 10 d after birth, newly born cells extend axons into the CA3 subfield of the hippocampus [9,12]. After the first week of maturation, they also receive depolarizing GABA inputs [39–44]. Toward the end of the second week, GABA inputs become progressively hyperpolarizing, and the adult-born neurons begin receiving functional glutamatergic depolarizing afferents [11,29,40,41], a process that occurs in parallel with the formation of dendritic spines.

On the basis of this developmental pattern, it seems likely that newborn neurons that are younger than 5 d are not influenced by learning because they lack afferent inputs and have not yet reached projection territories. Neurons that are in the window during which learning induces apoptosis should have received functional depolarizing GABA inputs, although their dendritic tree would still be poorly developed, and these newborn cells should not have reached their target area. Thus, in response to learning-driven depolarization, this imbalance between input and output activity may impede the survival of these cells and lead to their death. Older neurons that survive as a consequence of learning have a more developed dendritic tree that receives depolarizing GABA inputs and starts to have some glutamatergic ones. Furthermore, these newborn neurons have also reached the CA3 subfield. It is then likely that these newborn neurons that have reached a higher stage of maturation, with balanced input/output connections, can benefit from the pro-differentiating effects of the activation by GABA and glutamatergic inputs by learning [45].

Whether or not the newly born neurons whose survival is increased by learning participate in the memory process remains an open question. Although newborn neurons need several weeks before reaching full functional maturation (for review see [45]), they may participate in the processing of memory at immature stages due to their high plasticity level [46,47]. These peculiar properties may explain why immature neurons are responsive to life experiences within a critical time period [30]. Surviving newly born neurons having similar birthdates may induce the formation of functional neuronal assemblies in the CA3 subfield, and the resulting new circuits may store memory traces [48]. Alternatively, addition of these new circuits could encode the time of new memories [49]. However, recent studies have shown that although spatial behaviors preferentially activated new neurons in the dentate gyrus [50,51], this recruitment did not occur until they were at least 4 wk old [50]. Thus, if the neurons whose survival is increased by learning are not recruited by the ongoing behavior, they may support a subsequent learning experience. Additional investigations are required to determine whether adult-born neurons exert a functional role in memory formation before or after reaching complete maturity

Our observations also show that spatial learning is not onlybased upon the addition of new neurons or synaptic connections, but also upon regressive events that culminate in the removal of neurons from the cellular network of the adult central nervous system. An interplay between the addition and removal of adult-born neurons as a mechanism that sustains learned behavior has already been reported for adult songbirds [52,53]. Interestingly, our results show that relationships between learning, neurogenesis, and apoptosis are quite different in mammals and in birds. In the adult male canary, for example, neurogenesis is triggered by a wave of apoptosis of adult neurons within the higher vocal center [54]. The current interpretation of these processes is that the death of older neurons and their substitution by new ones allows the canaries to forget the song repertoire learned the previous year and replace it with a new one [52]. In mammals, during the encoding of new information, it is the apoptosis of younger neurons that facilitates the survival of older ones. As a consequence, whereas apoptosis in birds subserves the substitution of older learning for new, in mammals, apoptosis seems to allow the efficient adding up of new information.

In conclusion, our results show that spatial learning involves a mechanism very similar to the selective stabilization process observed during brain development, in which the production of new neurons is followed by an active selection of some and removal of others. As a consequence, spatial learning is not only based upon additive processes, ranging from synaptic strengthening to the formation of new synapses and new neurons, but also upon regressive phenomena, such as neuronal apoptosis. This epigenetic specification of networks by removal of neurons in the adult brain provides evidence of an additional mechanism contributing to the establishment of memory formation in mammals.

## Materials and Methods

### Water-maze training.

Three-month-old male Sprague-Dawley rats were tested in a water maze according to a previously described method [14]. Briefly, animals were tested 2 wk following their arrival. The apparatus consisted of a circular swimming pool built of white plastic (180-cm diameter, 60-cm height) filled with water (20 ± 1 °C) that has been made opaque by the addition of a nontoxic white cosmetic adjuvant. Before the start of the training, the animals were habituated to the pool for 2 d for 1 min/d. During training, the Learning group (L) was composed of animals that were required to locate the submerged platform, hidden 1.5 cm under the water in a fixed location, using the spatial cues available within the testing room. They were all tested for four trials per day (90 s with an intertrial interval of 30 s and beginning from three different start points that varied randomly each day). If an animal did not find the platform, it was set on it at the end of the trial. The time to reach the platform (latency in seconds) was collected using a video camera fixed to the ceiling of the room and connected to a computerized tracking system (Videotrack; Viewpoint, http://www.viewpoint.fr/en_EU/) located in an adjacent room that received the individual home cages of rats during testing. For the probe test (60 s), performances were assessed by the time spent in the target quadrant where the platform was previously located. For the cued test (90 s), performances were assessed by the latency to reach the visible platform located in a different quadrant than the one used for the nonvisible platform.

In the first experiment (Table S1), two control groups were used: a Control group (C) consisting of animals that were transferred to the testing room at the same time and with the same procedures as the learning group but that were not exposed to the water maze, and a Yoked group (Y), a control for the stress and motor activity associated with the water-maze training, composed of rats that were placed into the pool without the platform and were paired for the duration of the trial with the Learning animals. In the second experiment, three control groups were used: a Control group, a Yoked group, and an additional group of rats that were trained to find a visible platform (VP) in a fixed location. Animals in this group were all tested for four trials per day (90 s with an intertrial interval of 30 s and beginning from three different start points that varied randomly each day). Because in the first experiment the Yoked and Control groups did not differ for cell genesis or cell death, and because in the second experiment the Visual Platform, Yoked, and Control groups also did not differ, only the Control group was used for subsequent experiments. All experiments were performed in accordance with the European Union (86/609/EEC) and the French National Committee (87/848) recommendations. Detailed analysis of swim paths made to reach the platform location during the probe test was performed using the Wintrack software (see Protocol S1).

### Intracerebroventricular surgery.

Guide cannulae were implanted, according to a previously described method [55], above the rostral ventricle in order not to cause lesions in the hippocampus. Two weeks later, 6 μl (per infusion site) of vehicle (Ringer's solution with 1% DMSO) or of zVAD-fmk (at a concentration of 1 μg of zVAD/μl of vehicle; Calbiochem, http://www.emdbiosciences.com/html/CBC/home.html) [25] solutions were infused at a constant rate (3 μl/min) in naive or in trained animals immediately after the last trial of the 4th–6th days (batches 8 and 11) or of the 1st–3rd days (batch 9) of training.

### Thymidine analog injections.

Newly born cells were labeled by the incorporation of synthetic thymidine analog (XdU [where X represents Br, Cl, or I]; Table S1). Rats (batches 1, 3–8, and 11) were injected with BrdU (intraperitoneal). The Learning groups received one daily BrdU injection 30 min before the first trials or a single BrdU injection 3 or 4 d before the onset of training. Rats of the 11th experiment received a single injection of IdU and of CldU [27], respectively, 7 and 3 d before the onset of training, both at equimolar doses of 50 mg BrdU/kg. The control groups were injected with XdU within the same period.

### Immunohistochemistry.

Animals were sacrificed 1 d (batches 1, 2, and 11) or 3 h after daily training session (batches 3–7), or after the probe test (batches 8 and 9). Free-floating sections (50 μm) were processed in a standard immunohistochemical procedure in order to visualized BrdU (1/200; Dako, http://www.dako.com), IdU (1/1,000, BD PharMingen #347580; BD Biosciences, http://www.bdbiosciences.com), CldU (1/1,000; Accurate Chemical and Scientific Corporation, http://www.accuratechemical.com), Ki67 (1/200, Novocastra; Vision BioSystems, http://www.vision-bio.com), fractin (1/5,000, BD PharMingen #551527; BD Biosciences), HH3 (1/2,000, Cell Signaling #06–570; Upstate Biotechnology, http://www.upstate.com), and activated-caspase-3 (1/10,000, BD PharMingen #551150; BD Biosciences)[14,22,56]. Sections were counterstained with thionine in order to visualize pyknotic cells, characterized by a condensed nucleus of smaller size, and karyorrhexic cells displaying chromatin clumps. The number of immunoreactive (IR) cells throughout the entire granule and subgranular layers of the DG were estimated using the optical fractionator method [14].

To examine the phenotype of BrdU-IR cells, one in ten sections were incubated with BrdU antibodies (1/500; Accurate), which were revealed using a CY3–anti-rat antibody (1/1,000; Jackson Immunoresearch, http://www.jacksonimmuno.com). Sections were then incubated with anti-DCX antibodies (1/1,000; Santa Cruz Biotechnology, http://www.scbt.com), which were visualized with an Alexa-488 anti-goat IgG (1/1,000; Jackson). The percentage of BrdU-labeled cells expressing Dcx was determined throughout the DG using a confocal microscope with helium–neon and argon lasers (DMR TCSSP2AOBS; Leica, http://www.leica-microsystems.com). To estimate the total number of BrdU-labeled neurons, the percent of BrdU-IR cells co-labeled with Dcx was multiplied by the total number of BrdU-labeled cells.

### Electrophysiology.

Hippocampal slices (500 μm) were prepared as described previously [57] from vehicle- and zVAD -infused rats by an investigator blind to the treatments. Slices were submerged in an oxygenated artificial cerebrospinal fluid (ACSF) comprising (in mM): NaCl 123, KCl 2.5, Na_2_HPO_4_ 1, NaHCO_3_ 26.2, Cacl_2_ 2.4, MgCl_2_ 1.2, glucose 10, bicuculline 0.02 (pH 7.4; 295 mosmol.kg^−1^; room temperature). A concentric bipolar steel electrode was placed in the stratum radiatum to evoke (0.01 Hz) field excitatory postsynaptic potentials (fEPSPs) recorded with a glass electrode filled with ACSF. Data were collected with a multiclamp 700A (Axon Instruments, http://www.axon.com), filtered at 3 kHz, sampled at 10 kHz and analyzed offline using pClamp 9 (Axon Instruments). The initial slopes of the fEPSPs were measured from approximately 10%–40% of the rising phase. Paired-pulse ratio corresponds to the slope ratio of the second fEPSP to the first fEPSP.

### Statistical analysis.

All data (mean ± standard error of the mean) were analyzed by a Student *t*-test (two tailed) or by analysis of variance followed by Newman-Keuls test when necessary. Correlation analysis was performed using the Spearman test.

## Supporting Information

Figure S1 Correlations between the Performance in the Water Maze and Apoptotic Cell Death In order to characterize the relationship between learning and changes in cell death, we correlated the performance in the water maze (mean latency to find the platform after 3 to 6 d of training) with the number of fractin-IR cells or of pyknotic and karyorrhexic cells (Apoptotic cells). A significant correlation was found between the behavioral performances and cell death ([A] fractin-IR cells: *R* = −0.47, *p* < 0.001; [B] Apoptotic cells: *R* = −0.62, *p* < 0.001). In addition, the number of fractin-IR cells also correlated to the number of apoptotic cells ([C] *R* = 0.65, *p* < 0.001).
(22 KB PDF)

Figure S2 Influence of zVAD Infusion during the Early Phase of Learning on Spatial Learning Vehicle or zVAD was infused during the first 3 d of water-maze training (batch 9). Results show vehicle- and zVAD-infused animals did not differ in their latency to find the hidden platform during training ([A] *F*_1_,_16_ = 0.07, *p* > 0.05) nor for the time spent in the target quadrant during the probe test on the seventh day ([B] *t*_16_ =1.20, *p* > 0.05).
(19 KB PDF)

Figure S3 Effects of the Blockade of Caspases-Mediated Cell Death on Hippocampal Neurophysiological Response Field recordings performed in hippocampal slices (batch 10) revealed that zVAD did not alter the input–output relationship (*F*_6,30_ = 0.34, *p* > 0.05) nor short-term facilitation at CA3-CA1 synapses, indicating that excitatory synaptic transmission (*F*_6,30_ = 1.46, *p* > 0.05) was unaffected by zVAD.
(A) Example of CA1 field excitatory postsynaptic potentials (fEPSPs) obtained at different stimulus intensities.
(B) Input–output relationships in animals infused with zVAD (•) or vehicle (○).
(C) Example of short-term facilitation occurring when pairs of stimuli were applied 50 ms apart at different stimulus intensities.
(D) Facilitation (paired-pulse ratio) plotted as a function of stimulus strength.
(28 KB PDF)

Figure S4 Example of IdU- and CldU-Labeled Cells Cells having incorporated IdU or CldU were specifically revealed with BD #347580 (1/1,000^e^) or Accurate (1/1,000^e^) antibodies, respectively. There was no cross reactivity between antibodies. Scale bar indicates 10 μm.
GCL, granule cell layer.
(100 KB PDF)

Protocol S1 Detailed Analysis of Swim Paths during the Probe Test (30 KB DOC)

Table S1 Summary of the Schedule of Pharmacological Treatments and Water-Maze Training (16 KB PDF)

Table S2 Repartition of Cell Death within the Dentate Gyrus Learning-induced cell death occurred prominently in the subgranular layer where neuronal precursors reside. Cell death was much less in the granule cell layer composed mainly of mature neurons (*F*_3,28_ = 65.78, *p* < 0.001).
(12 KB PDF)

Table S3 Effect of Learning on Cell Death in the Different Regions of the Hippocampus Learning did not modify the number of fractin-IR cells and of pyknotic and karyorrhexic cells (Apoptotic cells) in the CA3 and CA1 subfield of the Ammon's Horn.
(12 KB PDF)

Table S4 Time Course of the Influence of Learning on the Number of BrdU-IR Cells Spatial learning had no effect on the number of newborn cells labeled with BrdU during the first 3 d of training (D1–D3, see Table S1). D, day.
(11 KB PDF)

Table S5 Influence of zVAD Infusion during the Late Phase of Learning on the Probe Test and the Cued Test During the probe test, zVAD treatment impaired all the indices used to measure the efficiency of the swim paths to reach the goal location (time to goal, Wishaw's index, and cumulative search error and path efficiency index). In contrast, during the cued test, zVAD treatment did not alter either visuomotor processes (average speed, and latency to reach the visible platform).
(11 KB PDF)

## Abbreviations

BrdU: 5-bromo-2′-deoxyuridine
CldU: 5-chloro-2′-deoxyuridine
Dcx: doublecortin
DG: dentate gyrus
fEPSP: field excitatory postsynaptic potential
IdU: 5-iodo-2′-deoxyuridine
IR: immunoreactive
zVAD: z-Val-Ala-Asp-fluoromethylketone
