# Supplementary material for: Spatial Learning Depends on Both the Addition and Removal of New Hippocampal Neurons
Source: PLoS Biol. 2007 Aug 7;5(8):e214. doi: 10.1371/journal.pbio.0050214 (PMC1939885; doi:10.1371/journal.pbio.0050214)
Supplement: Supplementary file 5 [file pbio.0050214.st001.pdf]

Batches	Experiments	Days of XdU injections in respect to training days (Dn)	XdU dose (mg/kg)	Groups size	Duration of training	Time of sacrifice in respect to the start of training
Results section:	***Specific phase of spatial learning induces apoptotic cell death in the dentate gyrus***					
1	Influence of spatial learning on cell death in the dentate gyrus	BrdU on D1-D4	50	Control = 7<br>Yoked = 6<br>Learning = 8	8 days	9th day
2	Influence of cue learning on cell death in the dentate gyrus			Control = 5<br>Yoked = 5<br>Learning = 5<br>Visible PF = 5	6 days	7th day
3	Influence of the different phases of spatial learning on cell death in the dentate gyrus	BrdU on D1-D3	50	Control = 8<br>Learning = 10	3 days	3rd day
4	“	BrdU on D1-D3	50	Control = 9<br>Learning = 15	4 days	4th day
5	“	BrdU on D1-D3	50	Control = 9<br>Learning = 14	5 days	5th day
6	“	BrdU on D1-D3	50	Control = 8<br>Learning = 13	6 days	6th day
Results section:	***Spatial learning promotes the death of newborn neurons within a certain time window***					
7a	Identification of the time window sensitive to learning-induced cell death	BrdU on - D3	100	Control = 8<br>Learning = 6	5 days	5th day
7b	“	BrdU on - D4	100	Control = 5<br>Learning = 5	5 days	5th day
Results section:	***Learning-induced apoptosis is critical for spatial memory***					
8	Influence of zVAD on spatial learning	BrdU on D1-D3	50	Control Veh = 9<br>Learning Veh = 12<br>Control zVAD = 10<br>Learning zVAD = 12	6 days +<br>1 day probe test<br>& cued test	7th day
9	Analysis of non specific effect of zVAD on spatial learning			Learning Veh = 8<br>Learning zVAD = 10	6 days +<br>1 day probe test	7th day
10	Analysis of non specific effect of zVAD on hippocampal neurophysiological responsiveness			Control Veh = 3<br>Control zVAD = 4		
Results section:	***Learning-induced increases in apoptosis, cell proliferation and survival of newborn neurons are interrelated processes***					
11	Analysis of relationship between learning-induced increase in cell survival and cell death	IdU on - D7<br>CldU on - D3	57.65<br>42.76	Control Veh = 5<br>Learning Veh = 9<br>Control zVAD = 6<br>Learning zVAD = 8	6 days	7th day
