# Supplementary material for: Spatial Learning Depends on Both the Addition and Removal of New Hippocampal Neurons
Source: PLoS Biol. 2007 Aug 7;5(8):e214. doi: 10.1371/journal.pbio.0050214 (PMC1939885; doi:10.1371/journal.pbio.0050214)
Supplement: Supplementary file 6 [file pbio.0050214.st002.pdf]

Zone	% Dying cells
Subgranular layer	74.87±5.25
Internal granule cell layer	9.90±3.04
Medial granule cell layer	6.96±4.26
External granule cell layer	8.27±3.51

Dupret et al – Supplementary Table S2
