# Supplementary material for: Spatial Learning Depends on Both the Addition and Removal of New Hippocampal Neurons
Source: PLoS Biol. 2007 Aug 7;5(8):e214. doi: 10.1371/journal.pbio.0050214 (PMC1939885; doi:10.1371/journal.pbio.0050214)
Supplement: Supplementary file 7 [file pbio.0050214.st003.pdf]

	CA3 subfield		CA1 subfield	
**Groups**	*Fractin-IR cells (Nb)*	*Apoptotic cells (Nb)*	*Fractin-IR cells (Nb)*	*Apoptotic cells (Nb)*
Control	5.71±2.97	47.14±13.04	15.71±2.97	81.42±9.61
Yoked	8.33±1.66	58.33±12.49	18.33±5.42	76.66±14.52
Learning	6.66±3.33	41.11±6.33	21.11±4.54	91.11±8.75

Dupret et al – Supplementary Table S3
