# Supplementary material for: Spatial Learning Depends on Both the Addition and Removal of New Hippocampal Neurons
Source: PLoS Biol. 2007 Aug 7;5(8):e214. doi: 10.1371/journal.pbio.0050214 (PMC1939885; doi:10.1371/journal.pbio.0050214)
Supplement: Supplementary file 8 [file pbio.0050214.st004.pdf]

Batches	Number of BrdU-IR cells		Statistical significance
	Control	Learning	
3	3412.50±238.05	3804.01±273.04	t_{16}=1.05, p>0.30
4	3306.66±391.18	3633.33±253.30	t_{22}=0.73, p>0.47
5	3131.11±327.24	3377.85±266.99	t_{21}=0.58, p>0.56
6	3933.75±279.01	4049.23±453.88	t_{19}=0.18, p>0.85

Dupret et al – Supplementary Table S4.
