# Supplementary material for: Spatial Learning Depends on Both the Addition and Removal of New Hippocampal Neurons
Source: PLoS Biol. 2007 Aug 7;5(8):e214. doi: 10.1371/journal.pbio.0050214 (PMC1939885; doi:10.1371/journal.pbio.0050214)
Supplement: Supplementary file 9 [file pbio.0050214.st005.pdf]

Behavioral parameters	Trained rats		Statistical significance
	Vehicle	zVAD	
**Probe Test**			
Time to goal (s)	11.59±1.86	24.44±4.52	t_{22}=-2.65, p<0.01
Wishaw's index (%)	43.87±8.36	23.84±5.35	t_{22}=2.41, p<0.05
Cumulative search error (m/s)	4.93±1.81	16.26±4.05	t_{22}=-3.12, p<0.01
Path efficiency index (%)	47.25±6.10	28.87±4.60	t_{22}=2.63, p<0.01
Average speed (m/s)	0.29±0.02	0.29±0.02	t_{22}=0.19, p>0.05
**Cued test**			
Latency to reach the visible platform (s)	6.49±0.66	9.03±0.99	t_{22}=-1.83, p>0.05

Dupret et al – Supplementary Table S5
