# Supplementary material for: Spatial Learning Depends on Both the Addition and Removal of New Hippocampal Neurons
Source: PLoS Biol. 2007 Aug 7;5(8):e214. doi: 10.1371/journal.pbio.0050214 (PMC1939885; doi:10.1371/journal.pbio.0050214)
Supplement: Supplementary file 10 [file pbio.0050214.sd001.doc]

**Supporting information**

**Protocol S1.** A detailed analysis of swim paths made to reach the platform location during the probe test (Table S5) was performed using the Wintrack software. The following parameters were examined: 1) time to reach the goal, 2) the Wishaw’s index corresponding to the percentage of path traveled within a straight corridor connecting the start and the goal, 3) the cumulative search error corresponding to the difference between observed cumulative distance and minimal cumulative distance. The cumulative distance to the goal is computed by multiplying the average distance to the goal by the time elapsed until reaching the goal; the minimal cumulative distance for an optimal swim path is estimated by dividing the squared airline distance from the release point to the goal by two times the animal's average swim speed (periods of inactivity are not considered for the computation, 4) path efficiency index corresponding to the percent of path traveled within a deviation by 15° or less of the direction of movement from the direction pointing to the goal. For all these analyses, all data points in the goal zone, as well as the periods of inactivity are not considered for computation. Spatial histograms of the animals’location were constructed by transforming each swim path in xy coordinates in function of time. Data surface were expressed using the Ratio Mode. For more detailed informations, see website <http://www.dpwolfer.ch/wintrack>.
